# COVID-19 and treatment with NSAIDs and corticosteroids: should we be limiting their use in the clinical setting?

**DOI:** 10.3332/ecancer.2020.1023

**Published:** 2020-03-30

**Authors:** Beth Russell, Charlotte Moss, Anne Rigg, Mieke Van Hemelrijck

**Affiliations:** 1 Translational Oncology and Urology Research, School of Cancer and Pharmaceutical Sciences, King’s College London, London SE1 9RT, UK; 2 Guy’s Cancer Centre, Guy’s and St Thomas’ NHS Foundation Trust, Great Maze Pond, London SE1 9RT, UK; * Denotes joint first authorship.

**Keywords:** COVID-19, SARS-CoV-2, non-steroidal anti-inflammatory drugs, corticosteroids

## Abstract

Given the current SARS-CoV-2 (COVID-19) pandemic, the availability of reliable information for clinicians and patients is paramount. There have been a number of reports stating that non-steroidal anti-inflammatory drugs (NSAIDs) and corticosteroids may exacerbate symptoms in COVID-19 patients. Therefore, this review aimed to collate information available in published articles to identify any evidence behind these claims with the aim of advising clinicians on how best to treat patients. This review found no published evidence for or against the use of NSAIDs in COVID-19 patients. Meanwhile, there appeared to be some evidence that corticosteroids may be beneficial if utilised in the early acute phase of infection, however, conflicting evidence from the World Health Organisation surrounding corticosteroid use in certain viral infections means this evidence is not conclusive. Given the current availability of literature, caution should be exercised until further evidence emerges surrounding the use of NSAIDs and corticosteroids in COVID-19 patients.

Across Europe, the incidence and mortality rates of COVID-19 or severe acute respiratory syndrome corona virus 2 (SARS-CoV-2) continue to rise dramatically. The disease is named due to the similarities to the SARS outbreak in China of 2002, which was also caused by a coronavirus. However, SARS-CoV-2 is thought to be caused by a novel coronavirus not seen before. Alongside uncertainty related to infection aetiology and outcomes, emerging concerns relate to the use of common non-steroidal anti-inflammatory drugs (NSAIDs) and corticosteroids. The Belgian Federal Agency for Medicines and Health Products released a statement on 16th March 2020 stating ‘It is well known that NSAIDs and corticosteroids can lead to serious complications’. Equally, a report by French Authorities suggested the use of ibuprofen in COVID-19 patients was detrimental to patient condition and recovery. NSAIDs are often used for the management of mild pain in cancer patients; hence, this topic is of particular importance to these patients. We, therefore, sought to gather information on the use of NSAIDs or corticosteroids and COVID-19, through systematic review of existing literature.

A search was conducted using Ovid MEDLINE and 13 studies were identified as suitable for inclusion [1–13]. Due to COVID-19’s novel status and disease similarities, studies relating to the 2002 SARS-CoV outbreak were also selected for review and these formed the majority of the literature. Crucially, this review did not identify any strong evidence for or against the use of ibuprofen during treatment of COVID-19 specifically.

One study within the review linked SARS-CoV to the downregulation of ACE2 [1]. This downregulation of ACE2 has previously been implicated in the loss of pulmonary function in SARS-CoV [1]. However, further data also suggested that ACE2 expression is increased through the use of ibuprofen, in diabetic patients and in those treated with angiotensin II type-I receptor blockers [2]. Consequently, it was suggested that increased expression of ACE2 in these co-morbid patients could facilitate infection with COVID-19.

The only other study to investigate NSAID was one which looked at indomethacin, which is commonly prescribed for treatment of gout and arthritis [11]. This study suggested that indomethacin exhibited potent antiviral activity against canine coronavirus *in vitro*, by dramatically inhibiting virus replication and protecting the host cell from virus-induced damage. Critically, this activity was also observed *in vivo* and against human SARS-CoV at a concentration dose of 1 mg/kg.

Most other included studies investigated the use of corticosteroids rather than NSAIDs during the treatment of coronaviruses [4, 5, 7–10, 12, 13]. Overall, we identified generally positive outcomes for the use of corticosteroids, particularly with reference to the SARS-CoV outbreak where they were widely used due to their known ability to modulate the inflammatory response. Various studies in humans noted that corticosteroids appeared effective in reducing immunopathological damage, but concerns centred around the promotion of viral rebound and association with adverse events (including acute respiratory distress syndrome). For example, a randomised controlled trial which measured viral load at regular intervals in non-intubated SARS-CoV cases found higher concentrations of viral RNA in week 2/3 of infection in those treated with corticosteroids compared to placebo [9]. Equally, a laboratory study which treated porcine respiratory coronavirus infected pigs with dexamethasone suggested that one or two doses of the corticosteroid in the acute phase of infection may effectively alleviate early pro-inflammatory response, but prolonged administration may play a role in enhancing viral replication [10]. Conversely, however, a separate Chinese study, which separated SARS-CoV patients into four treatment groups, identified early high dose steroids in combination with a quinolone produced the most favourable patient outcomes [5]. A similar mouse model study identified the N-protein of SARS-CoV as responsible for inducing pulmonary inflammatory reaction and acute lung injury which were related to the increase and imbalance of pro-inflammatory and anti-inflammatory cytokines [12]. Glucocorticoids, in the form of dexamethasone, effectively alleviated the pulmonary inflammatory reaction in these mice.

## Conclusion

In conclusion, the existing literature does not currently provide conclusive evidence for or against the use of NSAIDs in the treatment of COVID-19 patients, though there appears to be some evidence that corticosteroids may be beneficial if utilised in the early acute phase of infection [14]. However, it is important to note this is not specific to COVID-19. Indeed, one review stated that the WHO does not currently recommend corticosteroids in other viral diseases, like Dengue as the ‘glucocorticoid-mediated stimulation of the hypothalamic-pituitaryadrenal axis can also drive lymphocytopenia, or it may promote exaggerated pro-inflammatory responses that eventually cause a worsening of the pathogenic condition’. These are unprecedented times for the medical community and although evidence suggests a potential role for the use of NSAIDs and corticosteroids in COVID-19 treatment, caution should be exercised until further evidence, specific to this infection strain, emerges. The same guidance stands for cancer patients who are not advised to change their medication routine unless told otherwise by their doctor.

## Conflicts of interest

The authors have no conflicts of interest to declare.

## Funding declaration

This work was supported by Guy's and St Thomas' Charity.
